# Slovak parents’ mental health and socioeconomic changes during the COVID-19 pandemic

**DOI:** 10.3389/fpsyt.2022.934293

**Published:** 2022-08-18

**Authors:** Lenka Vargová, Gabriela Mikulášková, Denisa Fedáková, Martin Lačný, Jaroslava Babjáková, Martina Šlosáriková, Peter Babinčák, Ivan Ropovik, Matúš Adamkovič

**Affiliations:** ^1^Institute of Psychology, Faculty of Arts, University of Presov, Prešov, Slovakia; ^2^Instytut Psychologii, Wyższa Szkoła Humanitas, Humanitas University, Sosnowiec, Poland; ^3^Institute of Social Sciences of the Centre of Social and Psychological Sciences, Slovak Academy of Sciences, Bratislava, Slovakia; ^4^Faculty of Arts, Institute of Political Science, University of Presov, Prešov, Slovakia; ^5^Department of Preschool and Elementary Education and Psychology, Faculty of Education, University of Presov, Prešov, Slovakia; ^6^Faculty of Education, Institute for Research and Development of Education, Charles University, Prague, Czechia

**Keywords:** parents, mental health, depression, anxiety, stress, COVID-19, pandemic, economic situation

## Abstract

The changes in people’s mental health have become one of the hot topics during the COVID-19 pandemic. Parents have been said to be among the most vulnerable groups in terms of the imposed anti-pandemic measures. The present paper analyzes the trends in mental health indicators in a sample of Slovak parents (*N* = 363) who participated in four waves of data collection over a year and a half of the COVID-19 pandemic. The mental health indicators were represented by general levels of depression and anxiety as well as COVID-related stress and anxiety. While there were only minor changes in depression and anxiety, the dynamic in COVID-related stress and especially anxiety was more noteworthy. Besides some exceptions, the results hold even after controlling for the socioeconomic situation. The gender differences in the mental health trends were found to be negligible. Overall, we observed no substantial deterioration in the mental health indicators across the four waves of the study.

## Introduction

Since the beginning of the COVID-19 pandemic, its impact on mental health has been studied intensively [e.g., (1, 2)]. There have been worldwide measures put in place to curb the spread of coronavirus. However, these have disrupted the work and family-life routines of individuals. Social isolation, closure of educational institutions, financial insecurity, and changes in health and social care have impaired the lives of families, especially those with children (3). In particular, parents have been exposed to more stressful situations which have concerned them as well as their children (4). Parents have also experienced difficulties in taking care of homeschooled children during online teaching in addition to keeping down their job. This has made them vulnerable to financial insecurity, income decrease or job loss (5). Thus, the negative impact of COVID-19 appears to have been much more significant for parents compared to non-parents (6).

The objective economic situation of many families has worsened significantly as a result of the pandemic. There has been a global increase in unemployment (7); note that jobs most-at-risk are concentrated in lagging regions, (8) in addition to no-pay leave, reduced working hours (9), liquidity constraints, income shocks and the related decrease in consumption (10). The economic slowdown has exacerbated the pre-existing health and socio-economic inequalities as the pandemic has progressed (11). In Slovakia, the unemployment rate peaked in April 2021 at 8.8% (12). In spite efforts to provide economic aid, particularly for parents with children (13), there was notably low satisfaction with the pandemic support in Slovakia and Slovaks also reported serious household difficulties making ends meet and the fear of future financial worsening (14).

Various studies have confirmed an association between both objective and subjective economic status and mental health issues [e.g., (15–19)]. Therefore, it is likely that the economic changes during the course of the pandemic have affected mental health, especially among parents. Higher levels of depression, anxiety, stress, parental burnout, and worsened family relationships have been mainly observed in parents who have suffered socio-economic hardship, low-income families and families that have experienced multiple hardships or economic losses (20–25). In addition, the deterioration of mental health has been more notable in mothers as their work-life balance has been particularly affected by the anti-pandemic measures (26–28).

Parents’ struggles, such as an increased reliance on negative coping strategies (29) or mental health worsening (30), tend to get reflected in children’s at-home education, their well-being, emotional regulation or, in severe cases, maltreatment (31–34). While it is undeniable that the pandemic has caused tremendous socio-economic changes and disrupted the functioning of many families, general evidence of how families adapt in the long term is still lacking (21). There has only been one study published (35) concerning the mental health of Slovak parents. However, the study deals with a specific context and sample (i.e., parents of children with autistic spectrum disorder, predominantly mothers). Furthermore, only a few studies have dealt with specific COVID-related measures of mental health. In some of these studies, single items concerning worry, fear, stress, or COVID-related stressors have been used (36, 37). In other studies, COVID-related mental health scales have been used [e.g., COVID-related stress or anxiety; (38–41)]. However, to the best of our knowledge, there is a lack of studies that target the dynamic of COVID-related mental health constructs in a sample of parents.

In order to address these gaps, complex and especially longitudinal evidence is needed. In contrast to cross-sectional data, a longitudinal design means it is possible to study the dynamics of mental health indicators and subsequently help to understand the rate of change. The present study thus aims to analyze the changes in Slovak parents’ mental health over a year and a half of the COVID-19 pandemic, while also accounting for the effect of the objective, as well as subjective, economic situation. In particular, we will examine the trends will be examined in four mental health indicators out of which two are general (depression and anxiety), and the other two are COVID-related (COVID-related anxiety and COVID-related stress). The study focuses on a sample of parents from a representative sample of the Slovak population across four time points. The study also examines differences between mothers and fathers in these trends.

## Materials and methods

### Data collection and participants

A longitudinal design with four waves of online data collection (October 2020, December 2020, December 2021, and March 2022) was implemented. The first three waves were intentionally carried out during a period of lockdown or very strict measures. The fourth wave took place during a period of eased (almost non-existent) restrictive measures.

Before the first wave of data collection, the total number of positive cases in Slovakia since the beginning of the pandemic was approximately 10,000. By October 2020, when the first wave of data collection was carried out, the number of new daily cases had grown rapidly, and at the end of October, the total number of positive cases was almost 60,000. In December 2020, during the second wave of data collection, this number exceeded 150,000 (growing rapidly – at the end of December, it exceeded 180,000). In November and December 2021, after a relatively stable period, another increase of new positive cases emerged. During the third wave of data collection (December 2021) the total number of positive cases in Slovakia since the beginning of the pandemic had reached 840,000 (end of December). Up to March 2022, when the fourth data collection was carried out, this number had continued to grow rapidly. At the end of March, the total number of positive cases crossed 1,700,000. After that, the situation stabilized (42, 43). The vaccination rate in Slovakia was low at the time of the third and the fourth wave of data collection, and remains low even today. Vaccination against COVID-19 in Slovakia started at the end of 2020 (December 26), with priority given to at-risk groups. Vaccination for all people aged 16 + was launched in May 2021. By the end of the summer, approximately 40% of the population had been fully vaccinated and by the end of 2021, the vaccination rate had reached almost 50%. In early 2022, vaccination almost stopped due to lack of interest and now, in the summer of 2022, the vaccination rate is approximately 51% (42); Ministry of Health of the Slovak Republic). For more details about the pandemic situation in Slovakia please see this overview https://osf.io/vjmfd/.

The current sample (*N* = 363) is a subset of a representative sample (based on quota characteristics for gender, age, education, and region) of Slovak inhabitants and consists of parents who, at the time of the third wave, had (or were taking care of) at least one child younger than 18 years. The participants took part in all four waves of data collection. About 52% of the participants were women and the mean age of the sample at T1 was 45.4 years (SD = 10.8). For more details on the demographic characteristics see Table 1.

**TABLE 1:** Demographic characteristics of the participants.

Variable	Percentage or Mean ± SD
Gender (female)	51.79%
Age	45.43 ± 10.82
Partner status (married/in relationship)	78.79%
Education (university degree)	31.96%
Residence (urban)	63.36%
Economic status (employed)	69.42%
Number of children	1.84 ± 0.89

### Measures

The participants were asked about demographics, economic situation (personal and household income, number of household members).

The subjective socioeconomic status (SES) was measured using a visual ladder (MacArthur scale of subjective social status (44). The instruction describes that on the top of the ladder are people with the highest socioeconomic status whereas the bottom of the ladder represents those with the lowest socioeconomic status. The participants indicate where they would place themselves on the ladder using a 10-point scale (from 1 = the lowest socioeconomic status to 10 = the highest socioeconomic status).

Income was assessed as an equivalized total net household income per month. The equivalization was done using a slightly modified OECD formula (45). This assigns a coefficient of 1 to one adult member of the family, 0.5 to all other adult members, and 0.3 to all children living in the household (U18).

Depression was measured using a QIDS-16-SR (46) questionnaire. The items cover 9 depression symptoms according to the DSM-5 (e.g., depressed mood, loss of interest or pleasure, etc.). For each item, the participants expressed the degree of symptom severity during the last 2 weeks (from 1 to 4).

Anxiety was measured using the Generalized Anxiety Disorder Screener-7 [GAD-7; (47)*]* which assesses the severity of generalized anxiety disorder symptoms (e.g., feeling nervous, anxious or on edge). The participants evaluated how often they had been bothered by individual symptoms (from 1 = not at all to 4 = nearly every day) during the last 2 weeks.

Covid-related anxiety was measured using the Covid-related anxiety stress scale [items from PAS; (48)], and consecutively modified according to (49). The items are formulated as statements measuring anxiety related to COVID-19 (e.g., worries about catching COVID-19.) The participants answered on a 5-point Likert scale (from 1 = not at all to 5 = completely).

Covid-related stress was measured using the Covid-related stress scale [adapted from the COVIDiSTRESS survey; (50)]. The items are formulated as statements assessing the presence of concerns and difficulties in various areas possibly affected by the COVID-19 pandemic (e.g., concerns about the socio-economic situation, daily functioning, etc.). The participants answered on a 7-point Likert scale (1 = no concerns at all; 7 = great concerns).

### Statistical analysis

Given the longitudinal nature of the data, the four waves of data collection were combined and the respondents who participated in all of them were matched. Out of the general population, participants who indicated they have (or take care of) at least one child under 18 years of age were selected. This item was available in the third wave of data collection. The data were screened for improbable values and careless participants as part of general data wrangling for the purposes of the research project APVV-20-0319 “Behavioural aspects of COVID-19: Mapping the COVID-related behaviours, and psychological, social, and economic consequences of the pandemic”). The reliabilities of the scales were checked using the omega total coefficient. The estimated omega total coefficients ranged from 0.84 to 0.96. We proceeded with calculations of a sum score for each of the mental health constructs at each timepoint and divided it by the number of items forming the construct. For descriptive purposes, descriptive statistics were also calculated, and the correlation matrix examined. To examine the sensitivity of our design to a range of hypothetical effect sizes, we carried out a power analysis and found that, given the sample size of *N* = 363, alpha level of 0.05, and the usage of two-sided test, our design had 48% power to detect an effect of *r* = 0.10, 97% power to detect an effect of *r* = 0.2, and almost 100% power to detect correlations larger than 0.3. For the mental health indicators, as well as for the socioeconomic indicators, a trend line was visualized for each participant and an average trend line was plotted (without any smoothing). In order to answer the research question (i.e., how the indicators of parents’ mental health changed over the four time points), three unconstrained linear growth models were run for each mental health indicator. The first of the three models did not include any of the covariates. In terms of accounting for one’s socio-economic situation, a time-varying covariate represented by one’s subjective perception of SES and the objective economic situation was included in the second and the third model, respectively. From each estimated model, the latent intercept, latent slope, their variances and intercept-slope covariance, including the SEs and *p*-values of these parameters were extracted. The models were estimated using the MLR estimator (Maximum Likelihood with Robust standard errors) and the missing data were imputed using FIML (Full Information Maximum Likelihood). The analysis was performed in R, using the lavaan package, version 0.6-8 (51).

## Results

**FIGURE 1:**
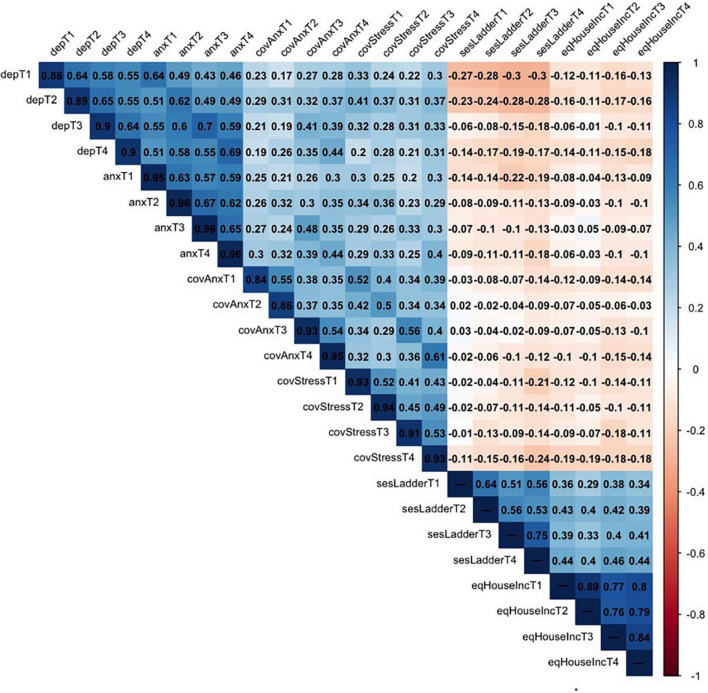
Correlation matrix and reliabilities. The reliablilities of the scales are presented on the diagonal.

**FIGURE 2:**
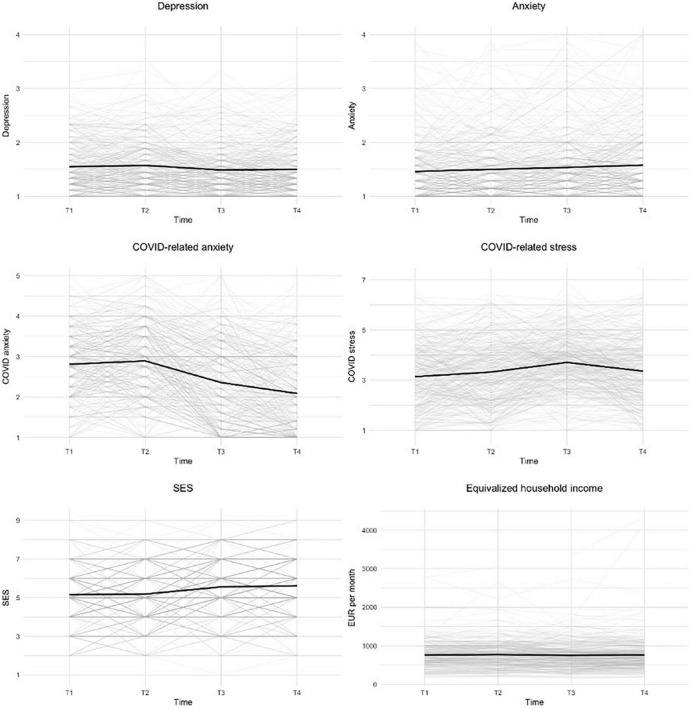
Visualization of the dynamic of mental health indicators and economic situation. Trend lines for each participant and average trend lines are plotted.

The descriptive statistics of the scales are available in Table 2. The correlation matrix with the diagonal representing the reliabilities of the scales is available in Figure 1. There are two things worth noting. Firstly, it is possible to see medium-to-high positive correlations between the mental health indicators. This indicates high comorbidity between the mental health issues and their (usually high) within-person stability in time. Secondly, there is a clear pattern of almost universally negative correlations between the socio-economic situation and mental health variables. The visualizations of the trend lines of mental health indicators, as well as participants’ socio-economic situation, are presented in Figure 2.

**TABLE 2:** Descriptive statistics and reliabilities.

	T1		T2		T3		T4			
Variable	Mean	SD	Mean	SD	Mean	SD	Mean	SD	Possible range	Omega total range
Depression	1.55	0.45	1.57	0.48	1.49	0.46	1.50	0.46	1–4	0.88–0.90
Anxiety	1.46	0.54	1.50	0.56	1.54	0.61	1.58	0.61	1–4	0.95–0.96
COVID anxiety	2.81	0.86	2.89	0.93	2.36	1.01	2.09	0.96	1–5	0.84–0.95
COVID stress	3.14	1.25	3.33	1.32	3.71	1.12	3.36	1.27	1–7	0.91–0.94
SES	5.15	1.46	5.18	1.49	5.56	1.49	5.62	1.52	1–10	–
Equivalized household income	761.71	370.11	772.81	382.41	752.77	375.23	763.59	451.71	–	–

In order to answer our research question (i.e., how the indicators of parents’ mental health changed over the four time points), three linear growth models were run for each mental health indicator.

The results of the linear growth models suggest the following patterns. There was a very small decrease observed in the level of depression (slope = −0.02, *p* = 0.006). This became more visible when controlling for SES (slope = −0.13, *p* < 0.001). On the other hand, a very small increase in anxiety was observed across the four time points (slope = 0.04, *p* < 0.001). This increase was more pronounced when the model controlled for income (slope = 0.11, *p* = 0.011). There was a substantial decrease in COVID-related anxiety observed in the model without any covariates (slope = −0.25, *p* < 0.001) as well as for the model that controlled for SES (slope = −0.23, *p* = 0.008). The decrease was slower for parents with a high initial level of COVID-anxiety (I-S covariances ranged from −0.08 to −0.09, ps < 0.001). Conversely, a notable increase was observed in COVID-related stress (slope = 0.11, *p* < 0.001). This was further amplified when a covariate was added into the model (slopes = 0.31 and 0.37; ps = 0.001 and.135; note the high SEs when income was controlled for in these models). For more detailed results, see Table 3.

**TABLE 3:** Results of the linear growth models.

Indicator	Covariate	Latent intercept	Latent intercept variance	Latent slope	Latent slope variance	Intercept - slope covariance
Depression	None	1.55***	0.14***	−0.02**	0.01*	–0.01
	SES	1.84***	0.13***	−0.13***	0.01*	–0.01
	Income	1.78***	0.14***	–0.05	0.01*	–0.01
Anxiety	None	1.46***	0.18***	0.04***	0.00	0.01
	SES	1.56***	0.18***	0.03	0.01	0.01
	Income	1.52***	0.18***	0.11*	0.00	0.01
COVID-anxiety	None	2.87***	0.50***	−0.25***	0.06***	−0.08**
	SES	2.91***	0.53***	−0.23**	0.07***	−0.09***
	Income	3.66***	0.52***	–0.18	0.06***	−0.09***
COVID-stress	None	3.22***	0.82***	0.11***	0.04	–0.06
	SES	3.15***	0.86***	0.31**	0.06**	–0.09
	Income	4.05***	0.82***	0.37	0.05*	–0.07

*p < 0.05, **p < 0.01, ***p < 0.001.

As an additional goal of the study, we examined the trends in the mental health outcomes separately for mothers and fathers. It was found that although mothers tend to score higher in most of the measures (models without a covariate), the group differences in changes in the mental health indicators over the course of the study were negligible. After accounting for SES, notable exceptions in this pattern occurred. In particular, depression in mothers decreased more sharply (slope difference = −0.10), anxiety decreased (slope difference -0.13), while the increase in COVID-related stress was less steep compared to fathers (slope difference = −0.11). After controlling for one’s household income, mothers got lower scores in depression (intercept difference = −0.17) and anxiety (intercept difference = 0.52) compared to fathers. Fathers’ scores for COVID-related anxiety (slope difference = 0.38) and COVID-related stress (slope difference = 0.69) rose compared to mothers. The observed increase was very steep, especially in the case of COVID-related stress. More detailed results are available in Table 4.

**TABLE 4:** Gender differences in (changes in) mental health indicators.

		Fathers			Mothers		
Indicator	Covariate	Latent intercept	Latent slope	I-S covariance	Latent intercept	Latent slope	I-S covariance
Depression	None	1.48	–0.03	–0.01	1.62	–0.01	–0.01
	SES	1.66	–0.07	–0.01	2.00	–0.17	0.00
	Income	1.92	–0.01	–0.01	1.75	–0.04	–0.01
Anxiety	None	1.34	0.03	0.00	1.56	0.05	0.01
	SES	1.34	0.11	0.00	1.73	–0.02	0.01
	Income	2.01	0.05	0.00	1.49	0.11	0.01
COVID-anxiety	None	2.71	–0.25	–0.06	3.02	–0.25	–0.10
	SES	2.65	–0.20	–0.08	3.13	–0.25	–0.10
	Income	3.28	0.08	–0.08	3.60	–0.30	–0.10
COVID-stress	None	3.01	0.12	–0.03	3.41	0.10	–0.08
	SES	2.84	0.37	–0.07	3.41	0.26	–0.11
	Income	2.70	0.79	–0.04	5.15	0.10	–0.09

## Discussion

Although the initial evidence has suggested a severe impact of the COVID-19 pandemic on mental health in the general population [e.g., (52–54), and parents in particular (23, 36, 55)], the present results have mostly indicated minor changes in parents’ mental health over the course of the study. The notable exceptions are a decrease in COVID-related anxiety and an increase in COVID-related stress after controlling for parents’ socio-economic situation.

At the beginning of the COVID-19 pandemic, there was little information about the virus or the situation in general available (56). In its initial phase, a worsening in mental health could be seen (57, 58). However, as both the amount of available information and people’s experience with the virus has increased, people have likely adapted to the situation and their mental health has stabilized or improved (59). On top of that, some people have ceased to think about COVID-19 being a serious threat or re-evaluated their perspective as a result of increased fake news and politicization of the situation (60). Concerns about health have played an important role in mental health during the pandemic (61). Although the population of parents has experienced more stress compared to non-parents (4), similar to the general population (62), the stress got lower as the perceived threat and severity of the situation decreased. In addition, the availability of vaccines and their efficiency has likely lowered the worries about health (63), helping to mitigate the negative impact of the pandemic situation on mental health.

With regard to the specific aspects of mental health measured in the current study, the results have shown a small decrease in the level of depression. Several studies have reported an increase in the depression rate due to the COVID-19 pandemic [e.g., (64–66)]. However, this increase was observed during the first few months of the pandemic. Fancourt et al. (67) have found that after depression peaked during the first few months, its level has tended to decrease and stabilize during later months. This is consistent with Kwong et al. (68) who observed no changes in the level of depression measured in April and May 2020, compared to the pre-pandemic level. The current results suggest that in the population of Slovak parents, reactive grief from an objective situation could have occurred instead of an increase in clinical depression. As such, the pandemic could be regarded as a temporary situation and people will learn how to cope with it. Although Martin et al. (69) have pointed out that people with higher pre-pandemic levels of depression are much more vulnerable to depression developing further, the study found no clear pattern suggesting that parents with higher initial levels of depression would get even worse or get better more slowly.

Valero-Moreno et al. (70) have found no increase in anxiety in the initial stages of the pandemic. Further evidence has also suggested a stabilization or even decline in the level of anxiety over time (67). In the present study, there was a small increase in anxiety observed across the four time points. Even though the results only indicate a minor impact of the pandemic on the changes in the level of anxiety, three important points can be discussed. Firstly, the data collection for the present study started in late 2020. While an increase in mental health issues seems to have occurred in the initial phase of the pandemic, there is no data to support or contradict this claim. Following other studies (57, 58, 67), it can be assumed that after the initial increase in the level of anxiety, a stabilizing trend is likely to have occurred. On the other hand, the pandemic situation during the first months was significantly better in Slovakia (the infection rate was one of the world’s lowest) compared to other countries. This might have caused a faster stabilization rate in the case of Slovakia.

Secondly, although stabilization was probably the most common mental health response to the pandemic, some subgroups may have also experienced worsening or improving mental health issues (71). As baseline data and information on pre-existing mental health status are not a part of the current study, interpretations should be made with some caution. While our data reflect a stabilizing trend, there is also a possibility that the levels of mental health issues found in the current study could reflect an increase which remained the same over time. More specifically, after the outbreak of the pandemic, an increase in mental health issues may have occurred and stayed at that higher level during our data collection. Alternatively, a worsening may have occurred in that subgroup of people with pre-existing mental health issues who were at higher risk of mental health deterioration (72). However, there is no data that could help clarify the possible heterogeneity of mental health responses to the pandemic in the current study (see limitations).

Thirdly, the measurement of mental health indicators should be taken into account. Many studies that have found modest changes in mental health during the COVID-19 pandemic assessed mental health using general measures [e.g., (71, 73, 74)]. However, it seems there is a qualitative difference between conventional mental health constructs and COVID-related mental health issues. This has been highlighted by Kubb and Foran (38) who discuss that findings concerning traditional measures of mental health should not be generalized to COVID-related mental health problems. The issue of COVID-related mental health and its consequences has been often neglected by researchers (75).

The present findings indicate a substantially higher dynamic of COVID-related mental health constructs compared to generally oriented depression or anxiety. In particular, the findings indicate that COVID-related anxiety started to decrease significantly after the first two waves of data collection. On the contrary, COVID-related stress increased over time, with its peak being observed in the third wave of data collection (late 2021). Stressors, their intensity as well as their qualitative aspects have changed during the pandemic (see (76, 77), and a fluctuation of stress related to the pandemic is thus natural (78). Consequently, there is a possibility of an interplay between COVID-related stress and COVID-related anxiety. In particular, the initial fear related to the pandemic can, through the inability to cope with stressors, develop into COVID-related anxiety. This is also supported by the strong bivariate relationships (*r*s > 0.50) between the constructs across all time points.

As COVID-related worries are determined by different stressors of different severity during different phases of the pandemic, COVID-related stress tends to get higher in the early phases of the pandemic or at its peak (78). In the current data, this is reflected by an increase in COVID-related stress in late 2021. This was when strict anti-pandemic measures were imposed in Slovakia and the infection and mortality rates were failing to decrease. More specifically, the infection rate and number of new daily cases were extremely high while the vaccination rate was low (see description of data collection). Furthermore, a new COVID-19 variant – Omicron – appeared in late 2021 and increased parents’ worries about their children’s vulnerability to this mutation (79). With an increased infection rate among children, parents were further exposed to the difficulties of ensuring care during the time of homeschooling, while also meeting other work-related needs. The available evidence has confirmed that children being in quarantine and parents’ difficulties in managing quarantine contributed to an increase in parental stress (33). With isolation from the wider family and teachers, parents were often left alone to take care of and educate their children. Childcare without sufficient external support is known to be stressful (80). The instability of the current pandemic situation and the associated difficulties in daily life has likely exacerbated the stress further (6).

We further analyzed the trends in mental health also after controlling for the subjective economic status and equivalized household income. The results indicate that a decrease in depression became more visible when the model controlled for the subjective SES. This result corresponds with the findings of Hoebel et al. (81) who have pointed out that the association between the objective economic situation and depressive symptoms is partially mediated through subjective SES for both men and women. The increase in general anxiety was more pronounced when the model controlled for income. For COVID-related anxiety, it seems that controlling for the socioeconomic situation does not affect its dynamics. On the other hand, a notable increase in COVID-related stress was amplified by both subjective SES and income. Various studies have confirmed the association between the economic situation and mental health issues during the initial phase of the pandemic (see (82). Indeed, parents tend to question their role and ability to take financial care of their family (83, 84).

In terms of the bivariate correlations between the constructs, it can be concluded that the relationships between the economic situation and mental health issues are almost uniformly negative. Valero-Moreno et al. (70) have noted a higher level of stress in parents at the beginning of the pandemic. The present data do not cover the initial phases of the pandemic and thus it is not known the extent to which parents’ level of stress at the end of the year 2020 differed from the levels in the early months. This raises the question as to whether the stabilization of COVID-related stress will follow the stabilization of the economic situation. This is especially after people have realized that economic worsening may not be as damaging as expected (85) or that it is reversible as the pandemic situation can improve [for example during the summer, even if the improvement is temporary; (86, 87)].

Given the aim of the study, gender differences can be discussed in the context of changes in depression, anxiety and stress separately. The results have confirmed that mothers tend to score higher in most measurements (models without covariates) and this corresponds to epidemiological data from 23 EU countries which all report a higher incidence of depression in women (88). On the other hand, the group differences between fathers and mothers in changes in mental health indicators during the pandemic were negligible although gender differences are observable when SES is taken into account. This may relate to socio-economic stratification across genders resulting from a variety of factors including differences in education choices, preferred job and industry, horizontal and vertical occupational gender segregation, work experience, number of hours worked, breaks in employment (such as for bearing and raising children), as well as gendered income disparity [see, e.g., (89, 90)]. The reduction in the symptoms of depression of mothers compared to fathers can be explained by the higher level of involvement in the education of their children during the pandemic period. The improvement in maternal depressive symptoms can be further explained by the higher presence of the father in the family (88) which is connected to better social support in their partnership (91). The results further showed that COVID-related anxiety and COVID-related stress increased in the group of fathers after controlling for household income. The increase was very sharp, especially in the case of COVID-related stress. It is likely that cultural expectations of the role of the father in the family in terms of providing income for the family will put more pressure on fathers.

### Limitations

The limitations of the present study can be interpreted on several levels such as limits related to the design of data collection, parental characteristics, social and personal context, and children’s characteristics. Participants were not asked about their pre-morbid status such as pre-existing mental health issues, medication or using supplements. Therefore, the evaluation of changes in people with pre-existing mental health issues [e.g., worsening of symptoms; recurrence, etc.; (92) is lacking in the present analyses]. Despite the longitudinal design of this study, the findings are limited to the changes that happened after the first several months of the pandemic and do not capture the initial dynamic in parents’ mental health (93). Furthermore, even though the data collection procedure was set to collect data from a representative sample of the Slovak population (based on quota characteristics for gender, age, education, and region), parents with lower SES (94) or parents below the poverty threshold are less likely to participate in the survey as they are known to have limited access to technology and internet. Moreover, specific financial concerns that could have affected the levels of (especially) stress were not assessed in detail. Future research could thus focus on these variables and examine which financial constraints have the strongest effect on parents’ mental health. At the same time, there was also no data on social support in the context of partnerships (80) nor on the level of resilience (95). These could both have affected the levels of stress and anxiety. It is possible that in some families the differences are caused by the parental role rather than by gender (96). Broader environmental and contextual factors could also have shaped the results. For instance, the strict anti-pandemic measures, including the obligatory quarantine, likely caused higher levels of mental health issues in the first two or possibly three waves of data collection. Moreover, factors like personal experience with the virus (e.g., the severity of the course of the infection, hospitalization, death of a close one, etc.) would have had an impact on one’s mental health but were not captured in the present study. For future research, it would be useful to take a look at social isolation due to the obligatory periods of quarantine. If a person has a positive attitude toward quarantine (considers it as protection from the virus), it might help them to cope with the situation better. The reduction in social activities has lowered the likelihood of getting infected. Thus, people that were more likely to adhere to the anti-pandemic measures, like social distancing, could have experienced less stress and fear about their health (97). Moreover, some people could have changed their lifestyle to handle the situation and stress related to the pandemic, restrictions, or lockdown (see (98). In addition, it could be of interest to examine if information about COVID-19 has been correctly presented. Clear and unbiased information and instructions can serve as a protective factor in alleviating mental health issues (65, 99, 100). Lastly, parents’ mental health could be determined by the characteristics of their children. In particular, the level of experienced stress in parents is associated with the perceived failure in taking care of their children, even more so if the child has special needs.

### Implications

The presented findings provide several fundamental proposals that could help improve health and social policies. We highly recommend paying more attention to parents’ mental health and especially to those who have a high baseline level of mental health issues. Moreover, parents’ mental health is mirrored in the well-being of their children. In terms of social policies, it seems to be of importance to pay extra attention to parents’ feelings and worries as their level of stress increases once they get concerned about their children’s safety, work-life balance and household income (20, 36, 84). Overall, the imposed anti-pandemic measures should also take into account the factors related to people’s mental health. In addition, governments should ensure easily accessible psychological or psychiatric aid to help alleviate the growing levels of stress. Social support programs, stress management training, and easy-to-implement interventions on emotional regulation strategies [e.g., reappraisal interventions; (101)] could positively affect parents’ ability to cope with the pandemic situation.

### Conclusion

Throughout the COVID-19 pandemic, societies and individuals have had to deal with a long-term crisis and intensive demands on safety and quality of life. This period of time has been accompanied by financial insecurity, job loss, or income reductions that could have occurred as a consequence of the pandemic. As parents’ mental health can have a wider impact, especially on their children (31), the current study aimed to look at the mental health of parents over a year and a half of the COVID-19 pandemic. In addition, differences between mothers and fathers were analyzed. The current results do not support the initial notions that the pandemic has a huge impact on mental health [e.g., (102–105)]. Indeed, the longitudinal evidence, including the presented study, has suggested that the deterioration of mental health has been small or negligible in both the general population (74) and population of parents (70). These results indicate that, in general, parents have adapted to the pandemic situation. In comparison to pre-pandemic times, a decrease in mental health issues has also been observed (64). The current study mostly found minor changes in parents’ mental health over the course of the study. Gender differences were also mostly negligible. There were some exceptions observed after controlling for the subjective and especially objective economic situation. However, more attention should be paid to COVID-related mental health issues and stressors which are likely to change over the course of the pandemic.

## Data availability statement

Data and the analytic script can be found at https://osf.io/epmt7/.

## Ethics statement

The studies involving human participants were reviewed and approved by Ethics Committee at the Faculty of Arts, University of Presov. The patients/participants provided their written informed consent to participate in this study.

## Author contributions

LV: data curation, conceptualization, investigation, writing – original draft, and writing – review and editing. GM: conceptualization, investigation, writing – original draft, and writing – review and editing. DF: conceptualization, investigation, supervision, writing – original draft, and writing – review and editing. ML: investigation, writing – original draft, and writing – review and editing. JB: conceptualization, writing – original draft, and writing – review and editing. MŠ: writing – original draft and writing – review and editing. PB: data curation, conceptualization, and writing – review and editing. IR: formal analysis, methodology, and writing – review and editing. MA: conceptualization, investigation, supervision, formal analysis, methodology, and writing – review and editing. All authors contributed to the article and approved the submitted version.

## Supplementary material

A detailed description of measures can be found at https://osf.io/bskr4/. A chronological overview of the pandemic situation and anti-pandemic measures in Slovakia is available at https://osf.io/vjmfd/. The full version of Table 3 is available at https://osf.io/ydq56.
